# Identification of Human Pathological Mitral Chordae Tendineae Using Polarization-sensitive Optical Coherence Tomography

**DOI:** 10.3390/s19030543

**Published:** 2019-01-28

**Authors:** Eusebio Real, José Manuel Icardo, Gaspar Fernández-Barreras, José Manuel Revuelta, Marta Calvo Díez, Alejandro Pontón, José Francisco Gutiérrez, José Miguel López Higuera, Olga María Conde

**Affiliations:** 1 Photonics Engineering Group, Department TEISA, University of Cantabria, 39005 Santander, Spain; miguel.lopezhiguera@unican.es; 2 Instituto de Investigación Marqués de Valdecilla (IDIVAL), 39011 Santander, Spain; fbarrerasg@gmail.com; 3 Department of Anatomy and Cell Biology, University of Cantabria, 39011 Santander, Spain; jose.icardo@unican.es; 4 Emeritus Professor of Surgery, University of Cantabria, 39005 Santander, Spain; josemanuel.revuelta@unican.es; 5 Cardiovascular Surgery Service, Marqués de Valdecilla University Hospital, 39011 Santander, Spain; martacalvodiez@hotmail.com (M.C.D.); aponton@humv.es (A.P.); josefrancisco.gutierrez@scsalud.es (J.F.G.); 6 Centro de Investigación Biomédica en Red - Bioengineering, Biomaterials and Nanomedicine (CIBER-BBN), 28029 Madrid, Spain

**Keywords:** polarization sensitive optical coherence tomography, birefringence, chordae tendineae, mitral valve

## Abstract

Defects of the mitral valve complex imply heart malfunction. The chordae tendineae (CTs) are tendinous strands connecting the mitral and tricuspid valve leaflets to the papillary muscles. These CTs are composed of organized, wavy collagen bundles, making them a strongly birefringent material. Disorder of the collagen structure due to different diseases (rheumatic, degenerative) implies the loss or reduction of tissue birefringence able to be characterized with Polarization Sensitive Optical Coherence Tomography (PS-OCT). PS-OCT is used to discriminate healthy from diseased chords, as the latter must be excised and replaced in clinical conventional interventions. PS-OCT allows to quantify birefringence reduction in human CTs affected by degenerative and rheumatic pathologies. This tissue optical property is proposed as a diagnostic marker for the identification of degradation of tendinous chords to guide intraoperative mitral valve surgery.

## 1. Introduction

Defects of the mitral valve imply heart malfunction and blood regurgitation, stressing the heart working conditions. Mitral regurgitation produces an increment of left ventricle pressure and hypertrophy to provide an efficient cardiac output. Degenerative and rheumatic mitral valve disease are among the principal pathologies affecting the mitral valve [1]. Mitral regurgitation is increasingly prevalent and, despite the fall in rheumatic disease, it is the second most common valvular lesion seen in adults in Europe [2]. 

Chordae tendineae are tendinous strands that connect the mitral and tricuspid valve leaflets to the papillary muscles [3]. The CTs are composed of a dense core of organized, wavy collagen bundles and of an outer spongiosa formed by loosely arranged collagen and covered by the endocardial endothelium [3,4]. Degenerative disease of the mitral valve (DDMV) affects the chordae tendineae [4,5]. When this occurs, the dense collagen tissue core evolves towards a loose tissue organization and the chordae may rupture [4]. In the case of the rheumatic disease, calcium aggregates deposit in the valve, causing thickening of the chordae and leaflets and inducing rigidity in both elements.

Significant mitral valve regurgitation requires surgical repair or prosthetic replacement. Surgical guidelines have been established to assess the best treatment on each scenario [6,7]. Mitral valve replacement with preservation of CTs and papillary muscles may maintain postoperative left ventricular function. Therefore, mitral valve repair is the preferred choice [8] as it reduces mortality and postoperative valve-related complications [9]. Different reconstructive surgical techniques are currently used. Neochord replacement with polytetrafluoroethylene (PTFE) is worldwide utilized with excellent surgical results [8,10,11]. In such surgical procedures, in situ and real time anatomical knowledge of CTs is crucial and an external assessment about the CT’s inner structural state is highly desirable, as no other perioperative diagnosis tool is available. Optical Coherence Tomography (OCT) and Polarization Sensitive Optical Coherence Tomography (PS-OCT) [12,13,14,15] are proposed to assess the structural conditions of the CTs. PS-OCT has been used to evaluate collagen in atherosclerotic plaques [16], changes in vertebral anulus fibrosus [17], altered retinal nerve fiber layer [18], cervix cancer [19], breast cancer detection [20], skin cancer [21] and others. PS-OCT is sensitive to the phase delay produced by birefringent tissues, such as the longitudinally ordered collagen fibers [13]. This makes the CTs a strongly birefringent material. This birefringence is proportional to the organization of the collagen within the chord, presenting measurable differences when this structure is modified due to different diseases [22]. PS-OCT is used to quantify birefringence differences in healthy and pathological human CTs from the mitral valve. This feature is used as a degradation marker in the case of degenerative and rheumatic chordal disease. The viability of this marker to identify degraded chords [23], in real time and avoiding histological studies, can turn PS-OCT into a useful and translatable in situ diagnostic tool during open surgery of the mitral valve.

## 2. Materials and Methods

### 2.1. Mitral Chordae Dpecimens

Human mitral CTs were obtained after surgical interventions at Marqués de Valdecilla University Hospital (Santander, Spain), after approval by “Comité Ético de Investigación Clínica de Cantabria”, and under DICUTEN project (code number 2017.009). Chord specimens were divided for this study into three different categories (Table 1): functional (25 specimens), degenerative (18 specimens) and rheumatic (18 specimens). Normal chordae are excised from valves with functional regurgitation without chordal affectation. Degenerative and rheumatic CTs were obtained from repaired or replaced mitral valves with these diseases. Specimens are preserved in 2% glutaraldehyde solution after excision, to be further analyzed with PS-OCT. Only marginal CTs were considered, being individually excised for every valve. All CTs present an extension longer than 10 mm. PS-OCT imaging was focused at the center of the chord where the mechanical structure becomes greatly impacted. 

### 2.2. Polarization Sensitive Optical Coherence Tomography

The OCT system used is an OCS1300SS (Thorlabs, Newton, NJ, USA) including a PSOCT-1300 Polarization Sensitive OCT module. This system is based on a frequency swept laser centered at 1325 nm, FWHM of 100 nm, providing B-scans of lateral scan size of 10 mm of length and penetration of 3 mm in air with a resolution of 25 µm lateral and 12 µm axial in air. C-scan of lateral dimensions 10 mm × 10 mm are obtained. With these scanning parameters the typical length and thickness of the chords are covered. From the whole C-scan, the B-scan at the maximum diameter of the chord is selected for the analysis. Each B-scan is comprised of 1024 A-scans of 512 pixels in depth. 

**Figure 1:**
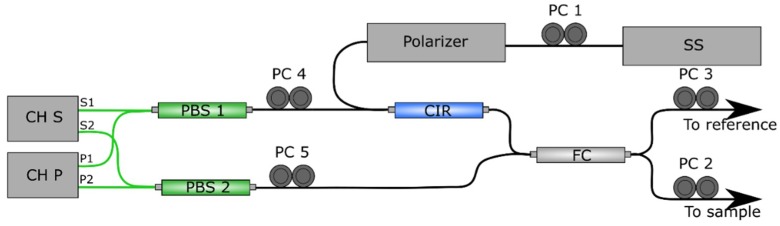
Schematic of the fiber-based setup PSOCT-1300 adapted from the manufacturer’s manual. Components are: balanced detectors (CH S and CH P), circulator (CIR), fiber coupler (FC), swept laser source (SS), manual fiber polarization controllers (PC numbered 1 to 5). Black lines indicate single-mode optical fiber (SMF), green lines indicate polarization-maintaining optical fiber (PMF).

The PS module generates birefringence induced phase-retardation images by detecting the vertical polarization state (CH S) and the horizontal polarization state (CH P) with two different detectors. The schematic of this setup is depicted in Figure 1.

The calibration procedure is stated by the manufacturer and achieved by manipulation of the manual fiber polarization controllers (PC) displayed on Figure 1, in combination with probing outputs used during calibration procedure. First, PC 1 is used to maximize power in the detector, achieving an adequate light source polarization state. PCs 3 to 5 are adjusted to minimize channel S and channel P deviation. PC 2 is used during measurement to control incident polarization state in the sample [24], compensating the effect of the probing single-mode optical fiber (SMF) and improving contrast in the phase retardation images.

The detection scheme consists of two balanced detectors, each detecting orthogonal polarization states CH S and CH P. The mathematical expression that models the system can be simplified to an amplitude modulation term and a phase dependent term [12]:

(1) $$\begin{array}{c} \mathrm{CH}\;S\propto K_{S}\;\sin\left(\delta /2\right)\\ \mathrm{CH}\;P\propto K_{P}\;\cos\left(\delta /2\right) \end{array}$$

where constants *K_S_* and *K_P_* account for source power and detector power efficiency in each detector and other possible variations in the different polarization detection scheme. Single-pass phase retardation, δ/2, can be obtained as [12,25]:

(2) $$\delta /2=\mathrm{atan}\left(\frac{K_{S}}{K_{P}}\frac{\mathrm{CH}\;S}{\mathrm{CH}\;P}\right)$$

When the response of both detectors is perfectly matched (*K_S_* = *K_P_*), the expression can be simplified as commonly seen in the literature [12,15,26,27]:

(3) $$\delta /2=\mathrm{atan}\left(\frac{\mathrm{CH}\;S}{\mathrm{CH}\;P}\right)$$

During the measurement procedure with the commercial OCT setup, the software provided by the manufacturer is implemented assuming that orthogonal polarization states in the detector are perfectly matched during the calibration procedure. For this reason, the output of the setup are cross-sectional images of round-trip phase retardation (0 deg ≤ δ ≤ 180 deg) according to Equation (3).

### 2.3. Birefringence with PS-OCT

Local estimate of birefringence can be calculated based on the rate of change of phase retardation with depth in images obtained with PS-OCT [26,27,28,29,30,31,32]. In the case of highly birefringent tissues, phase wrapping effect is produced when phase retardation is greater than 180 deg [33,34]. The accuracy of phase unwrapping is limited by SNR, which decreases with depth. Incident polarization misalignment contributes to SNR degradation, as well as other factors that increase error with depth, as the effect of diattenuation and change of the optical axis with depth [24]. These errors produce amplitude variations of the phase retardation with depth. 

Some authors have overcome this problem by using phase unwrapping algorithms that use Hilbert transform to eliminate amplitude variations in the calculation of phase retardation from the Stokes vector [24]. This allows a precise estimate of phase retardation, that is used to calculate tissue birefringence (Δ*n*) as the phase increment at a given penetration [24]. However, the accuracy of birefringence estimation based on phase increments relays on the quality of original OCT data, and incremental accumulation of errors in local phase imply accumulation of error in the estimate of birefringence. Under laboratory conditions, this can be controlled, but for systems intended to be used within the surgery room without interfering conventional clinical practices, calibration and usage must be simplified.

Here, birefringence is estimated without the unwrapping step, based on the work of other authors [13,17,35]. Being *λ*_0_ the central wavelength of the source, the estimated birefringence, Δ*n*, is computed by measuring phase retardation increment (Δ*δ*) given a certain penetration (Δ*z*):

(4) $$\Delta n=\frac{\Delta \delta \;\lambda_{0}}{2\cdot 360\;\Delta z}$$

Schoenenberger et al. [35] used Equation (3) to calculate phase retardation. Then, birefringence is computed by measuring Δδ given a fixed penetration before phase wrapping occurs in the tissue. De Boer et al. [13] use images obtained computing amplitude differences of orthogonal detected channels instead of phase retardation depicted on Equation (3). Here, in order to compute birefringence, Δz is measured as the distance between adjacent bands. 

**Figure 2:**
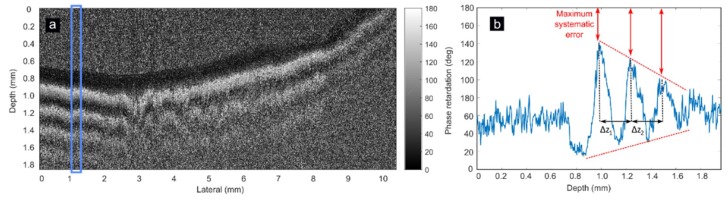
PS-OCT phase retardation image of one mitral chord specimen measured longitudinally (**a**). The average of A-scans marked with blue rectangle is shown in (**b**). A decay of phase amplitude is seen within depth (red dashed lines). Difference between 180 deg and empirical curve amplitude is indicative of maximum systematic error (red arrows). The period between phase retardation peaks (Δ*z*_1_ and Δ*z*_2_) is constant within depth (black arrows), not being affected by amplitude variations of the phase retardation.

In this work, the commercial OCT setup obtains phase retardation images according to Equation (3), representing phase retardation between 0 deg and 180 deg (Figure 2a). Due to the factors explained previously, measured phase retardance will not reach these values. Δn is obtained via Equation (4), measuring the period (Δ*z*) between fringes in the phase retardation image, corresponding with Δ*δ* = 360 deg. This way, considering only the period between fringes [17], local amplitude variations of phase and reduced SNR in depth do not affect as strongly as in other works (Figure 2b). By measuring the optical-versus-physical thickness of a thin slice [36], the average refractive index of mitral chords is measured as *n* = 1.389 [37].

### 2.4. Image Processing 

**Figure 3:**
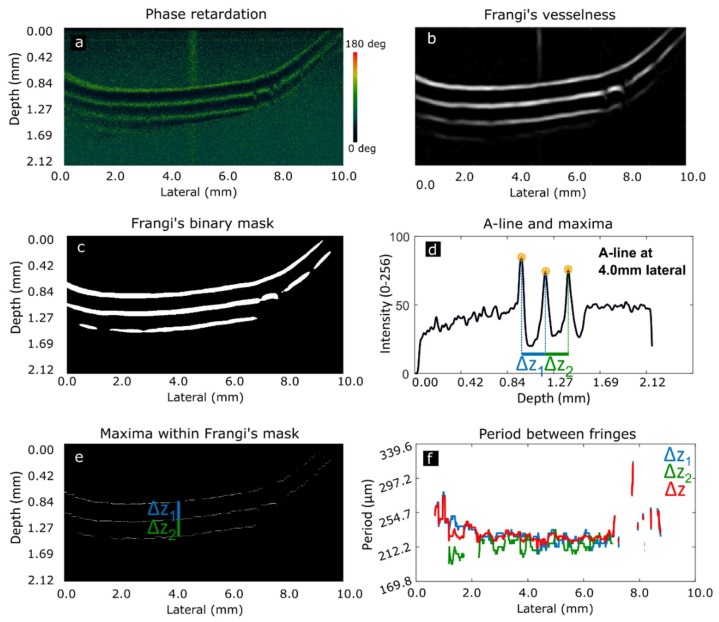
Phase retardation image obtained with PS-OCT module (**a**). Fringes highlighted with Frangi’s vesselness (**b**). Fringes binary mask (**c**). Average of 10 A-scans at 4.0 mm lateral scan range showing identification of maxima and periods (**d**). Δ*z*_1_ represents period between the first two fringes, Δ*z*_2_ between second and third fringes (**e**). Period obtained for every A-scan and average period Δ*z* (**f**). Absence of period in some points of is due to lack of period detection at these lateral scan positions.

PS-OCT images are color coded between 0 and 180 deg (Figure 3a). This color map is converted to gray scale preserving phase information. A soft Gaussian filter (σ = 3) is applied to reduce speckle noise and Frangi’s vesselness algorithm [38,39,40] is used to identify fringes in the image (Figure 3b), creating a binary mask (Figure 3c). This algorithm is based on the Hessian matrix of the image, used to highlight high level features that present long shape, such as fringes. The binary mask is used to improve maxima detection on the next step. A-scans are averaged with 10 neighbors to detect local maxima (Figure 3d). The distance between adjacent maxima are the measured periods Δz_1_ and Δz_2_. This procedure is applied on every A-scan, providing a clean visualization of maxima in the phase retardation images (Figure 3e). This allows calculation of periods Δz_1_ and Δz_2_ for the whole length of the chord (Figure 3f). Birefringence is finally computed according to Equation (3) for every A-scan, considering the average period, Δ*z*, as the mean value of Δ*z*_1_ and Δ*z*_2_. To reduce possible misdetections, periods are not computed in the first and last 50 pixels of every B-scan. A-scans lacking birefringence are not considered for the estimation of the mean birefringence, although they are contemplated for diagnostic purposes. On the other hand, if periods are only present on less than 10% of A-scans of the same B-scan, birefringence is also not computed because this indicates that the 90% of the length of the chord exhibit disorders in its structure.

### 2.5. Scanning Electron Microscopy

Scanning Electron Microscopy (SEM) images are obtained after PS-OCT imaging to assess the real degradation condition of the specimens under analysis. This technique allows visualization of large tissue areas as well as small details of the collagen organization. For SEM, selected chordae were excised longitudinally, and the fragments were dehydrated in graded acetone, dried by the critical point method, coated with gold and observed with an Inspect S microscope (FEI Company, Hillsboro, OR, USA) working at 15–20 KV, following routine procedures. Two representative specimens are analyzed for each category: functional, degenerative and rheumatic.

## 3. Results and Discussion

**Figure 4:**
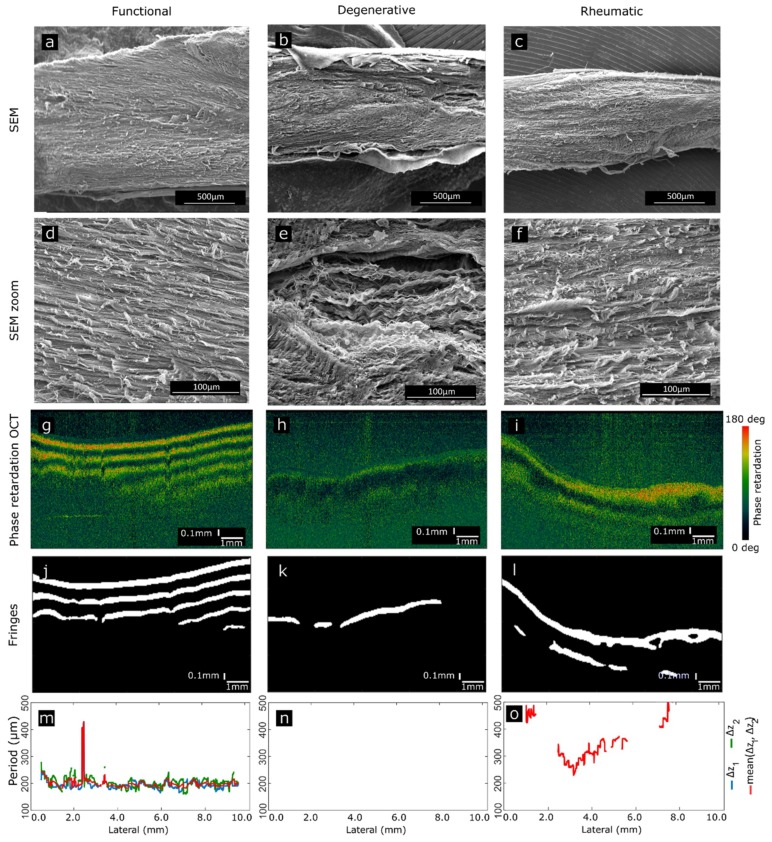
Comparison of three specimens belonging to each category. SEM has been used to assess the real composition and structure of the specimens. SEM images display long extensions of the chords (**a**,**b**,**c**), as well as zoomed regions (**d**,**e**,**f**) exhibiting specific peculiarities of each category. PS-OCT B-scans of the same chords (**g**,**h**,**i**) exhibit differences in phase retardation. Fringes are highlighted (**j**,**k**,**l**) as a step to compute the period (**m**,**n**,**o**) as the final step to estimate birefringence.

CTs of the three categories (functional, degenerative and rheumatic) have been analyzed with SEM. Functional CTs exhibit homogeneous collagen distribution and high density of collagen along the entire chordal fragment (Figure 4a,d). 

This is translated into phase retardation OCT B-scans as uniform birefringence patterns, presenting strongly defined fringes that preserve size and phase along the length of the chordae (Figure 4g,j). The period detected as a step previous to birefringence estimation is stable along the CT (Figure 4m).

Degenerative CTs show decreased homogeneity and lower density of collagen fibers (Figure 4b), with degradation in some areas. This is seen as the development of spaces between the collagen fibers, structural disorder and presence of torn and fray fibers (Figure 4e). The loss of the ordered healthy structure is translated into the phase retardation B-scans as either a loss of the fringes or as a reduction of birefringence (Figure 4h,k) being the shown example a specimen where no period can be retrieved (Figure 4n).

**Figure 5:**
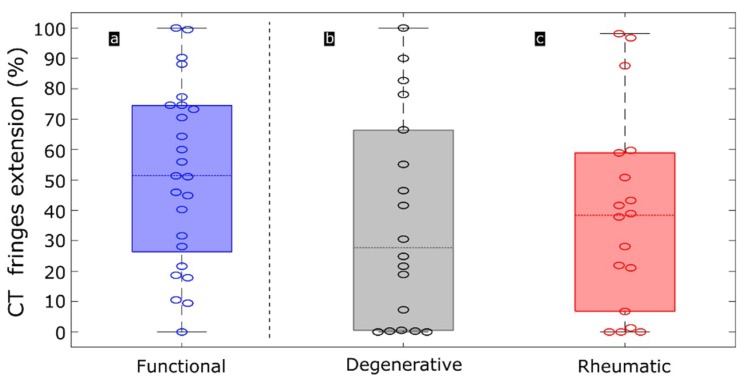
Boxplots representing the extension of periodic fringes along CTs of the three categories: functional (blue), degenerative (grey) and rheumatic (red). Extension is computed as a percentage of the B-scan length with A-scans exhibiting fringes. Dashed horizontal lines represent median of each category, this is, half of the observations is situated above this line and the other half is situated below this line. Boxes represent variation within 25% to 75% percentile. Black lines represent 5% to 95% of observations. Circles represent individual CTs.

Rheumatic CTs present thick collagen bundles tightly packed (Figure 4c,f). The close attachment of collagen bundles alters the typical and healthy looseness present in functional chords, reducing birefringence in PS-OCT or completely loosing this feature in cases of severe stiffness (Figure 4i). These observations agree with previous histopathological findings [3,4,5,22]. This is manifested as a lack of periodic fringes in some of the regions of the CT (Figure 4l), or as a difference in the period of the fringes along the CT, which translates into different birefringence values (Figure 4o).It should be stressed that in both, rheumatic and degenerative diseases, degradation is not uniform along the chordal length. This is seen both in SEM images and in PS-OCT phase-retardation patterns and evidenced in the period of the fringes. Depending on the chordae and due to expected tissue variability, the fringe pattern may disappear completely, or be present only in some regions of the specimens. Absence of fringes can be an indicative of a general pathologic status of the CT. Figure 5 shows the extension of the area of the B-scan where fringes appear with respect to the total length of the chord. This metric is used to discuss the three categories. In the case of functional CTs (Figure 5a), half of the A-scans present fringes in 51.45% of the CT length or more (Figure 5a, blue dashed line). In the case of degenerative CTs, half of the A-scans present fringes in less than 27.73% (Figure 5b, dashed line) and less than 38.37% of the extension in the case of rheumatic CTs (Figure 5c, dashed line). The presence of fringes in a large portion of the CT extension is related with a healthy chord whereas an absence of fringes, or its presence in a reduced extension, is related with a pathologic chord.

The presence of CTs with birefringence values in the degenerative and rheumatic categories is due to the heterogenous evolution of the pathology in the valve complex because this degradation process is progressive. On the other side, the lack of birefringence in some chords considered functional represents normal variation in valves that a priori present functional chords but affected by mitral insufficiency in a minor extension.

**Figure 6:**
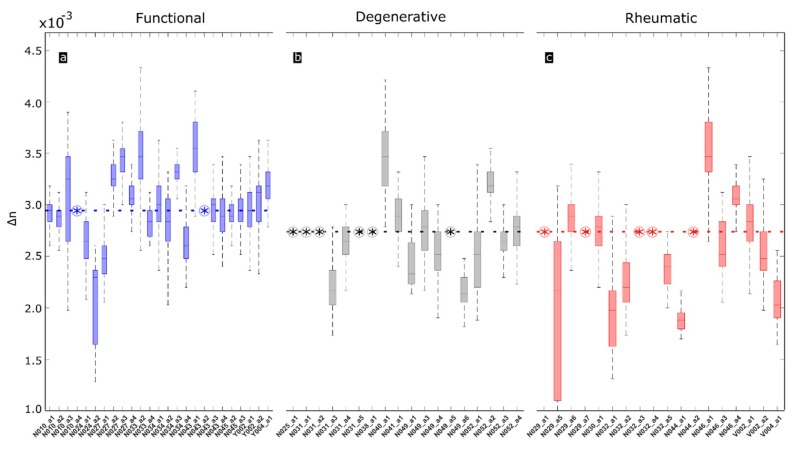
Boxplots representing birefringence value along individual CT specimens of the three categories: functional (blue), degenerative (grey) and rheumatic (red). Dashed horizontal lines represent median of each category. Encircled asterisks represent samples without birefringence. Boxes represent variation within 25% to 75% percentile. Black lines represent 5% to 95% of observations. Horizontal labels represent individual specimens accounting for number of patient (N) and number of chord (a).

The period between fringes and hence, birefringence, has been obtained for the entire length of the CTs. A-scans not presenting fringes are not considered to obtain the median value. Figure 6 displays the values of birefringence for each individual chord along its length. Each boxplot refers to each individual chord. The values of birefringence along the same CT are not constant as it could be expected with real specimens. Functional CTs (Figure 6a, blue) present higher birefringence values along the whole length of the CT. Degenerative (Figure 6b, grey) and rheumatic (Figure 6c, red) CTs, if they are birefringent, they present lower birefringence values when compared with functional CTs. 

An indicative of depth homogeneity of tissue could be addressed evaluating the difference among Δ*z*_1_ and Δ*z*_2_. This variation is, in average, of 2.22 % for functional specimens, 0.24% for degenerative and 4.15 % for rheumatic when compared with the average period of each category. This means that there is not much difference between periods, what allows considering the tissue as homogeneous in the region of two periods. 

**Figure 7:**
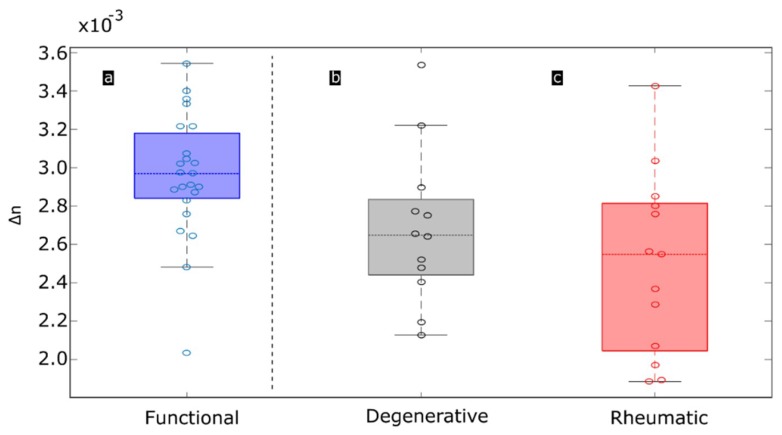
Boxplots representing birefringence of each of the three categories analyzed: functional CTs (blue), degenerative CTs (grey), rheumatic CTs (red). Circles represent observations. Black dashed line represents separation between healthy and pathological categories.

The median birefringence value is obtained for every CT specimen and represented for the three categories in Figure 6. As observed in Figure 7a (blue), functional CTs exhibit higher birefringence, with a median value of 2.9·10^−3^. Degenerative CTs (Figure 7b, grey) present lower birefringence, with a median value of 2.6·10^−3^. Rheumatic CTs (Figure 7c, red) present an even lower median birefringence value, being of 2.5·10^−3^. 

The birefringence values obtained in this work are comparable to those obtained with OCT in other tissues with similar content of collagen to mitral chordae, such as porcine tendons [24] and bovine tendon [13] (Table 2). Birefringence of human mitral chords have been studied by Whittaker et al. [22]. These authors used the Sénarmont’s method at 546 nm applying imbibition analysis to characterize the form birefringence of the material. Values obtained with this technique are affected by the imbibition media, however, they are considered in this work as they are obtained on the same tissue and the birefringence values are in the same range. Also, these authors used birefringence values to differentiate control CTs from pathological CTs (proteoglycan infiltration).

The goal for clinical practice is to discriminate functional from pathological (degenerative and rheumatic) chords to remove and repair the latter. As observed in Figure 7, functional and pathological categories can be discriminated. A statistical analysis of significance with Student’s t-test test at 5% significance level has been performed. Functional gives a p-value of 0.027 and 0.0022 when compared against degenerative and rheumatic categories respectively.

## 4. Conclusions

Human mitral CTs have been analyzed ex vivo with PS-OCT, and birefringence has been quantified for functional, degenerative and rheumatic chordae. SEM analysis has been used as a gold-standard for assessment of the collagen structure, linking the modifications of the structural organization of collagen with the total loss or the partial reduction of birefringence patterns. This application supports the expansion of PS-OCT functionality to the analysis and diagnosis of different tissues and pathologies [41], also in cardiovascular diseases [42]. Further developments, based on novel setups like eccentric-core fiber OCT [43] and assisted by real time processing methods based on computer Graphics Processing Unit (GPU) [44], will impulse translation to the surgery room and inclusion within common surgical practices.

Combined SEM and PS-OCT studies indicate that modifications of the collagen structure of the CTs can be related with the reduction or suppression of the birefringence-induced fringes, being the lack of this parameter an indicative of pathological disease. Birefringence computed along the entire length of the chordae is more uniform in functional specimens but exhibits strong variations along the same chordae in rheumatic and degenerative samples. This can be indicative that collagen structural alterations may occur in different degrees and extension, being dependent on pathological severity and current degradation stages. Tissue inhomogeneities will also appear as lack of fringes and, therefore, birefringence. The present results agree with structural studies in degenerative CTs [4]. The median birefringence of each chordae has also been obtained, showing higher values in the case of the functional chordae. The methodology used implies assumption of homogeneous tissue. When this condition is not fulfilled it is seen as a reduction or loss of birefringence, what helps for tissue classification. Complete lack of birefringence, or reduction of this parameter when quantified, appears as a potential marker for identification and quantification of pathology in individual CTs in the surgery room under intraoperative conditions. Some individual CTs, classified as functional revealed a lack of birefringence and hence, a possible degradation undetected by surgeons experienced eyes. This marker may be useful during mitral valve surgery, helping the surgeon decide the best procedure based on precise information obtained for every individual chord instead of a global visual perception. This can allow taking decisions comprising excision or repair of individual chordae or a complete valve replacement when chordae degradation is severe. Ongoing works will be aimed to find out how the birefringence parameter is correlated with the mechanical behavior of chordae [45], to predict performance and viability under load conditions.

## Figures and Tables

**Table 1:** Patient code divided in categories and number of CT specimens excised from each patient.

	Functional	Degenerative	Rheumatic
	N010 (4)	N025 (1)	N029 (4)
	N024 (2)	N031 (5)	N030 (1)
	N027 (4)	N038 (1)	N032 (5)
	N033 (2)	N040 (1)	N044 (2)
	N034 (4)	N041 (1)	N046 (3)
	N043 (4)	N049 (5)	V002 (2)
	N045 (2)	N052 (4)	V004 (1)
	Y002 (2)		
	Y004 (1)		
Total	9 (25)	7 (18)	7 (18)

Patient code (number of CTs per patient).

**Table 2:** Birefringence value for tendinous tissue obtained by different authors.

Author	Tissue	Method	Birefringence
This work	Human chordae tendineae	PS-OCT (Proposed)	1.9·10^−3^ (pathological) to 3.6·10^−3^ (functional)
De Boer et al. [13]	Bovine tendon	PS-OCT	3.7·10^−3^
Chin et al. [24]	Porcine tendon	PS-OCT	4.0·10^−3^
Whittaker et al. [22]	Human chordae tendineae	Sénarmont’s method	3.8·10^−3^ (pathological) to 4.7·10^−3^ (functional) (*)

(*) Obtained from form birefringence curves in imbibition medium of *n* = 1.33.
